# Novel therapies in breast cancer: what is new from ASCO 2008

**DOI:** 10.1186/1756-8722-1-16

**Published:** 2008-10-01

**Authors:** David Chu, Janice Lu

**Affiliations:** 1 Division of Medical Oncology, Department of Medicine, State University of New York at Stony Brook, Stony Brook, New York, USA

## Abstract

**Introduction:**

Breast cancer is the most common female cancer and the second most common cause of female cancer-related deaths in the United States. World-wide, more than one million women will be diagnosed with breast cancer annually. In 2007, more than 175,000 women were diagnosed with breast cancer in the United States. However, deaths due to breast cancer have decreased in the recent years in part because of improved screening techniques, surgical interventions, understanding of the pathogenesis of the disease, and utilization of traditional chemotherapies in a more efficacious manner. One of the more exciting areas of improvement in the treatment of breast cancer is the entrance of novel therapies now available to oncologists. In the field of cancer therapeutics, the area of targeted and biologic therapies has been progressing at a rapid rate, particularly in the treatment of breast cancer.

Since the advent of imatinib for the successful treatment of chronic myelogenous leukemia in the 2001, clinicians have been searching for comparable therapies that could be as efficacious and as tolerable. In order for targeted therapies to be effective, the agent must be able to inhibit critical regulatory pathways which promote tumor cell growth and proliferation. The targets must be identifiable, quantifiable and capable of being interrupted.

In the field of breast cancer, two advances in targeted therapy have led to great strides in the understanding and treatment of breast cancer, namely hormonal therapy for estrogen positive receptor breast cancer and antibodies directed towards the inhibition of human epidermal growth factor receptor (HER)2. These advances have revolutionized the understanding and the treatment strategies for breast cancer. Building upon these successes, a host of novel agents are currently being investigated and used in clinical trials that will hopefully prove to be as fruitful. This review will focus on novel therapies in the field of breast cancer with a focus on metastatic breast cancer (MBC) and updates from the recent annual ASCO meeting and contains a summary of the results.

## Novel Her-2/EGFR directed therapies

Treatment options for patients with breast cancer were traditionally based on cytotoxic chemotherapy but now include therapies directed towards identifiable targets which sustain tumor proliferation. Often these targeted therapies are more efficacious and at the same time less toxic than traditional regimens. The epidermal growth factor receptor (EGFR) is a transmembrane receptor with tyrosine kinase activity. The EGFR family includes HER1 (EGFR-1), HER2, HER3, and HER4. Mutations in this pathway lead to dysregulation in tumor cell proliferation and differentiation which makes this pathway an attractive target for biologic therapies. Led by the success of trastuzumab's HER2 blocking capabilities in breast cancer, EGFR inhibition with an emphasis on HER2 inhibition continues to be an area of focus in the treatment of breast cancer patients. A plethora of new agents directed towards the EGFR and HER2 pathway have been introduced and continue to demonstrate promising results.  The results are summarized in Table 1.  Cancer statistics can be found in [1].

**Table 1:** Summary of targets, toxicity, and evaluation at ASCO 2008 of novel agents in advanced breast cancer.

Agent	Target	Toxicity	Evaluation at ASCO
Lapatinib	EGFR/HER2	Diarrhea, rash, nausea, vomiting	-monotherapy in inflammatory BC
			-c/w trastuzumab
			-c/w bevacizumab

HKI-272	Pan HER	Diarrhea, nausea	n/a

Trastuzumab DM-1	HER2	Transaminitis, fatigue, thrombocytopenia, anemia, neuropathy	-monotherapy refractory to trastuzumab

Pertuzumab	HER2	Diarrhea, pain, nausea, vomiting, mucositis	-c/w trastuzumab

Tanespimycin	HSP 90	Fatigue, diarrhea, dizziness, headache	-c/w trastuzumab

Cetuximab	EGFR	Rash, diarrhea, nausea, vomiting	-c/w carboplatin in triple negative BC
			-c/w irinotecan

Bevacizumab	VEGF	Hypertension, proteinuria, bleeding, thromboembolism	-c/w lapatinib
			-c/w docetaxel
			-c/w nab-paclitaxel

Gefitinib	EFGR	Rash, diarrhea, nausea, vomiting	-c/w anastrazole

RAD001	mTOR	Stomatitis, fatigue, anorexia, diarrhea, headache, rash	-c/w anastrazole
			-c/w paclitaxel and trastuzumab
			-c/w navelbine and trastuzumab

Pazopanib	VEGFR, PDGFR, C-kit	Diarrhea, rash, nausea	-c/w lapatinib

Sunitinib	VEGFR, PDGFR, C-kit	Mucositis, fatigue, nausea, diarrhea	n/a

Axitinib	VEGFR 1,2, PDGFR, C-kit	Diarrhea, nausea, alopecia, stomatitis	n/a

C1311	Topoisomerase II	Neutropenia	-monotherapy in refractory BC

Pemetrexed	Anti-folate	Myelosuppression, anemia	-1^st ^line monotherapy in advanced BC

Larotaxel	Cytotoxic	Neutropenia, fatigue	-c/w trastuzumab

Orataxel	Cytotoxic	Neutropenia, fatigue, peripheral neuropathy	-monotherapy in taxane resistant BC

Abbreviations: ASCO; American Society of Clinical Oncology; EGFR, epidermal growth factor receptor; HER, human epidermal receptor; BC, breast cancer; c/w, combination with; n/a, not applicable; HSP, heat shock protein; VEGF, vascular endothelial growth factor; mTOR, mammalian target of rapamycin; VEGFR, vascular endothelial growth factor receptor; PDGFR, platelet derived growth factor receptor

### Tyrosine kinase inhibitors

#### Lapatanib

Lapatinib is an oral small-molecule dual inhibitor of the tyrosine kinase domain of both epidermal growth factor receptor (EGFR) and HER2/neu (ErbB-2). It was approved in March of 2007 for use in patients with advanced, refractory MBC in conjunction with capecitabine. Its initial testing was in HER2 positive MBC patients who experienced disease progression while receiving trastuzumab. In a phase II open-label multi-center study involving HER2 positive MBC patients refractory to trastuzumab, 80 patients were treated with lapatinib monotherapy at 1500 mg daily.[2] The overall response rate (ORR) was 8%, 14% of patients achieved stable disease and 22% of patients were free of progression. Adverse events with lapatinib were tolerable with anorexia, nausea, rash, vomiting, diarrhea, and weight loss being the most common events. Cardiotoxicity was not significantly observed. A second phase II study confirmed lapatinib's activity in HER2 positive MBC patients in the first-line setting.[3] In this study, 60 patients were randomized to either lapatinib 1500 mg daily or 500 mg twice daily. The ORR was similar in both groups: 28% in the 1500 mg daily group and 29% in the 500 mg twice daily group. There were no grade 3 or 4 adverse events. These two phase II studies led the way for lapatinib to be investigated in further clinical trials.

At ASCO 2008, lapatinib monotherapy was evaluated in patients with HER2 positive relapsed/refractory inflammatory breast cancer.[4] In this study, 126 HER2 positive patients with inflammatory breast cancer refractory to anthracyclines, taxanes, and traztuzumab were treated with continuous lapatinib monotherapy at 1500 mg daily. Preliminary data demonstrated an estimated ORR of 40%. The most frequent toxicities were diarrhea and skin rash. It was concluded that lapatinib montherapy is active in the treatment of relapsed/refractoryy HER2 positive inflammatory breast cancer where currently only a few effective therapies are available.

Lapatinib has also been successfully combined with chemotherapy in breast cancer patients. In an open-label study, patients with HER2 positive locally advanced and MBC who experienced disease progression after treatment with regimens that included an anthracycline, a taxane, and trastuzumab were randomly assigned to receive either combination lapatinib 1250 mg daily plus capecitabine 2000 mg daily or capecitabine 2500 mg daily alone.[5] The median time to progression (TTP) was 8.4 months in the combination group as compared with 4.4 months in the capecitabine monotherapy group. This improvement was achieved without an increase in serious grade 3/4 events. Again, cardiotoxicity was not a significant event. This trial led to its first approval for use in MBC patients.

Lapatinib was further tested in combination therapy when it was evaluated in conjuction with taxanes. In a phase III randomized double-blind study of 580 patients, lapatinib 1500 mg daily combined with paclitaxel 175 mg/m2 was compared with paclitaxel 175 mg/m2 alone as first-line treatment for patients with MBC irrespective of HER2 status.[6] The ORR was 35% vs 25% in favor of the combined group. However, TTP and overall survival (OS) were not significantly different between the two arms except in a subgroup of patients with HER2 positive advanced breast cancer. As expected, there was a significantly greater toxicity profile in the combination group over the paclitaxel monotherapy group with alopecia, nausea, vomiting, rash and diarrhea being the most common events.

A highly anticipated study was presented at ASCO 2008 involving heavily pretreated HER2 positive MBC patients progressing on trastuzumab therapy who were treated with lapatinib alone or in combination with trastuzumab.[7] In the study, 296 patients who were previously treated and suffered disease progression on trastuzumab therapy were randomized to either lapatinib 1000 mg daily plus trastuzumab 2 mg/kg weekly or lapatinib 1500 mg daily alone. The combination group achieved a significant improvement in progression free survival (PFS) (12 weeks vs 8.4 weeks) and clinical benefit rate (CBR) (25.2% vs 13.2%). However the differences in OS and ORR were not statistically significant. Both treatments were well tolerated with an asymptomatic decline in LVEF occurring in 5% of the patients in the combination arm and in 2% of the patients in the lapatinib monotherapy arm. This was the largest study using the combination of these two agents and the first to show synergy of the agents in a randomized setting.

At ASCO 2008, lapatinib was also combined with bevacizumab in a phase II single arm study evaluating 31 patients with heavily pretreated HER2 positive MBC.[8] Lapatinib was administered at a dose of 1500 mg daily and bevacizumab was administered at 10 mg/kg iv q 2 weeks. The study revealed that at 12 weeks PFS rate was 62%, and at 24 weeks the CBR was 56%. The combination of these two agents was tolerable with the most commonly reported adverse events being diarrhea, muscle pain, fatigue, nausea and vomiting. The trial continues to accrue, and updated results are pending. The implications for this combination are attractive in that the study showed promising activity in this group of patients without the increased cardiotoxicity that is faced when bevacizumab is combined with trastuzumab.

#### HKI-272

HKI-272 is an irreversible orally active pan-HER receptor tyrosine kinase inhibitor with potential anti-neoplastic activity. It binds to the HER2 receptor irreversibly thus reducing autophosphorylation in cells by targeting a cysteine residue in the ATP-binding pocket of the receptor which ultimately decreases tumor cell proliferation.[9] It is highly active against HER2 overexpressing human breast cancer cell lines in vitro.[9] It also inhibits the EGFR kinase and proliferation of EGFR-dependent cells.[9] It has been evaluated in a phase II study involving advanced HER2 positive breast cancer patients.[10] In this open label phase 2 study, 49 advanced HER2 positive breast cancer patients were divided into two treatment arms. The first arm included HER2 positive breast cancer patients who were previously treated with trastuzumab and the second arm included advanced HER2 positive breast cancer patients with no prior trastuzumab treatment. Tumor response and progression-free survival data continues to be gathered, but out of 32 evaluable patients, 6 patients achieved a confirmed partial response with additional patients achieving an unconfirmed partial response. Diarrhea and nausea were the major adverse effects noted in the study. Early preliminary data shows that daily administration is generally tolerable and has antitumor activity in patients with HER2 positive advanced breast cancer.

A phase I/II of HKI-272 in combination with trastuzumab in patients with advanced breast cancer has currently reached accrual and is awaiting data analysis.[11] It is also currently being evaluated in phase I/II studies in combination with paclitaxel as well as in combination with vinorelbine in patients with advanced breast cancer.[11] These investigations appear promising and may allow for further treatment options for patients with advanced HER2 positive breast cancers.

### Monoclonal antibodies

#### Trastuzumab DM-1

Trastuzumab DM-1 is a first in-class HER2 antibody drug conjugate which is designed to increase the potency antibody-directed therapy. Trastuzumab is combined with DM-1, a highly potent anti-microtubule agent derived from the fungal toxin maytansine.[12] At ASCO 2008, two phase I studies were presented for its use in HER2 positive advanced MBC. In the first study, 24 patients with HER2 positive MBC who had progressed on trastuzumab therapy received 156 doses of trastuzumab DM-1 at six different dose levels including 0.3 mg/kg, 0.6 mg/kg, 1.2 mg/kg, 2.4 mg/kg, 3.6 mg/kg and 4.8 mg/kg administered IV q 3 weeks.[13] 6 of 16 patients given doses at 2.4 mg/kg and 3.6 mg/kg achieved a PR and 5 additional patients achieved stable disease ongoing after 130 to 260 days. Adverse events included transaminase elevations, thrombocytopenia, fatigue, anemia and neuropathy. Cardiac toxicity was not observed. It was concluded that the maximal tolerated dose and recommended dose for phase II trials should be 3.6 mg/kg IV q 3 weeks as it is a tolerable and manageable dosing schedule. In the second phase I study, trastuzumab DM-1 was administered to HER2 positive MBC who had progressed on trastuzumab therapy given IV on a weekly basis in a dose-escalated fashion.[14] 7 patients were given trastuzumab DM-1 weekly at three different dose levels including 1.2 mg/kg, 1.6 mg/kg and 2.0 mg/kg and at the time of the presentation 4 patients achieved an unconfirmed PR. Adverse events included thrombocytopenia, fatigue, transaminase elevations, and headache. Again, cardiotoxicity was not observed. It was concluded that high-grade toxicities were minimal and dose escalation will continue until a maximal tolerated dose is achieved. These initial studies will hopefully be the spring board for launching this promising agent for treatment of trastuzumab resistant MBC patients.

#### Pertuzumab

Pertuzumab is a humanized monoclonal antibody that binds to the specific dimerization epitope of HER2 sterically blocking heterodimerization of HER2 thereby inhibiting intracellular signaling. Initial phase II studies of pertuzumab in MBC patients showed that pertuzumab was safe and well tolerated but had limited efficacy in this group of patients.[15] At ASCO 2008, an update on a phase II single-arm study of trastuzumab at 2 mg/kg q week or 6 mg/kg q 3 weeks combined with pertuzumab 420 mg q 3 weeks in patients with HER2 positive MBC who progressed during trastuzumab therapy was presented.[16] Initial results showed that out of 33 evaluable patients, an ORR was seen in 6 patients with one person achieving a CR and 5 patients achieving a PR. Furthermore, 7 patients achieved stable disease after 6 months, and 10 patients achieved stable disease in less than 6 months. The adverse effects of this combination included diarrhea, pain, nausea, vomiting and mucositis. This study suggests potential anti-tumor activity of pertuzumab in combination with trastuzumab in patients who are refractory to trastuzumab. The combination was well tolerated, and further studies with pertuzumab are ongoing.

### Hsp90 inhibitors

#### Tanespimycin

Heat shock protein 90 (Hsp9) is a molecular chaperone for various signaling proteins that promote cancer proliferation and resistance. Tanespimycin (17-AAG) is an ansamycin antibiotic that binds to Hsp90 and induces the degradation of proteins that require this chaperone thereby inducing tumor cell regression. Initial studies showed that this agent reduced ErbB2 levels and inhibited proliferation of trastuzumab resistant breast tumor cells.[17] This made it a potential agent in trastuzumab resistant HER2 positive patients. At 2008 ASCO, a trial combined tanespimycin 450 mg/m2 weekly and trastuzumab at standard dose in HER2 positive MBC refractory to trastuzumab therapy.[18] Of the 21 evaluable patients the ORR was found to be 24%, and the CBR was found to be 57%. 5 patients achieved a confirmed PR, 5 patients achieved stable disease, and 2 patients had a measurable response with a decrease in tumor markers. Toxicities that are common to cytotoxic chemotherapies such as alopecia, myelosuppression and neuropathy were not observed. The most predominant side effects were fatigue, diarrhea, dizziness and headache. It was concluded that the combination of tanespimycin and trastuzumab was active in HER2 positive MBC who progressed on trastuzumab based therapies with a relatively safe toxicity profile. These results suggest further investigation of tanespimycin in the treatment of HER2 over-expressed MBC patients.

### EGFR inhibitors

#### Cetuximab

Cetuximab is a human-mouse chimeric monoclonal antibody that competitively binds to EGFR to inhibit dimerization. It is currently approved for the treatment of both colorectal cancer and head and neck cancer. Its use in breast cancer has been evaluated in a phase II study of weekly irinotecan and carboplatinum with or without cetuximab in patients with MBC.[19] ORR was significantly improved with the addition of cetuximab than with irinotecan and carboplatinum alone (39% vs 19%). As expected the toxicity in the triple drug therapy group was increased but tolerable.

At ASCO 2008, an update of the TBCRC 001 trial was presented.[20] In this phase II multi-center randomized study in patients with metastatic triple negative (basal-like) breast cancer, 102 patients were randomized to either carboplatin AUC 2 plus cetuximab 250 mg/m2 weekly or carboplatin alone. The results revealed that in patients included in the carboplatin monotherapy arm 6% achieved a PR, 4% achieved stable disease, and the clinical benefit rate was 10%. In the combination arm, the ORR was 18%, 9% of patients had stable disease, and the clinical benefit rate was 27%. Although most patients progressed rapidly owing to the aggressive nature of the disease, it was concluded that single agent carboplatin had minimal activity in this type of MBC while the combination of carboplatin and cetuximab did show significantly improved anti-tumor activity.

Also presented at ASCO 2008 was a trial of cetuximab in combination with irinotecan in a phase II study in patients with MBC previously treated with an anthracycline or a taxane-based therapy.[21] In this study, 19 patients were treated with cetuximab 250 mg/m2 weekly and irinotecan 80 mg/m2, and the results showed that the ORR was 11% with one patient achieving a PR and 1 patient achieving a CR. One patient had stable disease for 11 cycles. The toxicity profile was well tolerated with dermatologic toxicities being the most common. It was concluded that although the combination therapy was well tolerated, the combination had minimal activity in this group of pretreated patients.

## Anti-angiogenesis agents

The vascular endothelial growth factor family of glycoproteins are ligands for receptor tyrosine kinases for which when overexpressed are thought to be essential for tumor growth and angiogenesis. Angiogenesis is necessary for cancer growth, invasion and metastasis. Several therapeutic agents have been developed which target this pathway and have already been incorporated into standard treatment regimens for numerous solid organ cancers. Breast cancer continues to be an area of interest for the use of these agents particularly in the metastatic setting.

### Bevacizumab

Bevacizumab is a humanized monoclonal antibody which inhibits all isoforms of vascular endothelial growth factor (VEGF). It has proven to have activity in combination with other chemotherapies in the treatment of several malignancies including lung, colorectal, renal and breast cancer. It initially gained attention for the treatment of breast cancer in a phase I/II dose escalation study of patients with MBC who had progressed on previous therapy.[22] In this study, 75 patients were treated with bevacizumab at a dose of 3 mg/kg, 10 mg/kg or 20 mg/kg in a bi-weekly basis. The ORR ranged from 6.7–17% in all patients. In the group of patients that were treated with 10 mg/kg, the ORR was 12%. This led the way for bevacizumab to be evaluated in further studies for the treatment of breast cancer.

Bevacizumab has been evaluated in combination with chemotherapies known to be efficacious in MBC. In a phase III randomized clinical trial of chemo-naive MBC patients coordinated by the Eastern Cooperative Oncology Group (ECOG) E2100, paclitaxel 90 mg/m2 in combination with bevacizumab 10 mg/kg was compared to paclitaxel alone.[23] The patients in the combination arm achieved a significantly longer PFS compared to the paclitaxel monotherapy arm (11.4 months vs 6.11 months) and a significantly increased ORR (30% vs 14%). However, the study failed to show a significant difference in OS. In a randomized phase III trial, bevacizumab was combined with capecitabine and compared to capecitabine alone in patients with previously treated MBC.[24] The study revealed that the combination group achieved a significantly greater ORR when compared with the capecitabine monotherapy group (19.8% vs 9.1%), but it failed to show a significant difference in OS or PFS.

At ASCO 2008, bevacizumab was further evaluated in combination therapy. In addition to the previous study involving combination therapy with lapatinib[8], bevacizumab was assessed in combination with docetaxel in a phase III randomized double blind placebo controlled study in locally recurrent or MBC referred to as the AVADO trial.[25] 736 patients from 24 different countries were treated with the combination of bevacizumab at high (15 mg/kg) and low dose (7.5 mg/kg) plus docetaxel 100 mg/m2 or docetaxel alone, and the results showed that PFS was statistically significantly superior for both bevacizumab containing arms compared to the docetaxel alone arm with a hazard ratio of 0.69 in the low-dose bevacizumab arm and 0.61 in the high-dose bevacizumab arm. ORR was also superior in both combination arms (55% in the low dose bevacizumab arm and 63% in the high dose bevacizumab arm) relative to docetaxel alone (44%). As the data was too immature at the time of the presentation, OS could not be assessed. Adverse events were increased in a limited fashion in both combination arms when compared to placebo, but the combination of docetaxel and bevacizumab did not reveal any new safety concerns.

At ASCO 2008, bevacizumab was also evaluated in combination with nab-paclitaxel for MBC patients in the first-line setting.[26] In this multi-center, open-label phase II study, 41 patients were treated with weekly nab-paclitaxel 125 mg/m2 in combination with bevacizumab 10 mg/kg as first-line therapy, and the results demonstrated an ORR of 30%. Stable disease at > 16 weeks was achieved in 22% of patients, and the median PFS was 9.2 months. The most common adverse events were neutropenia, anemia and peripheral neuropathy. It was concluded that the combination of nab-paclitaxel and bevacizumab is a tolerable combination with potential activity in MBC patients in the first-line setting.

Other studies presented at ASCO 2008 evaluated bevacizumab's safety and feasibility in combination with several current first-line therapies. In an ECOG coordinated trial labeled E2104, bevacizumab was evaluated in a phase II feasibility trial incorporating it into dose-dense doxorubicin and cyclophosphamide (AC) followed by paclitaxel (T) in patients with lymph-node positive breast cancer.[24] The primary endpoint was the incidence of clinically apparent cardiac dysfunction, and preliminary data suggests that the incorporation of bevacizumab into dose dense AC→T is a feasible and promising combination.

A second multi-center phase II double-blind randomized trial investigated the safety of bevacizumab at 7.5 mg/kg and 15 mg/kg in combination with docetaxel, doxorubicin and cyclophosphamide (TAC) for patients with stage II or III breast cancer in the neoadjuvant setting.[27] Of the 37 evaluable post surgical patients, the ORR was 95% including 59% of patients achieving a clinical CR and 35% of patients achieving a clinical PR. Data is currently being compiled and evaluated to detect a difference between patients that received and did not receive bevacizumab, but the preliminary data for this combination shows potential.

Stemming from the success of bevacizumab in combination with paclitaxel in E2001, a large, open-label single-arm study was presented which would analyze the safety and tolerability of bevacizumab in combination with taxane-based therapy for patients with locally recurrent or MBC.[28] This study will attempt to further elucidate the safety profile of bevacizumab with taxane-based therapy in a broader patient population by including patients in the community setting with plans to accrue more than 2,300 patients from 50 countries. The studies presented at ASCO 2008 will hopefully be the initial stages of incorporating bevacizumab into current standards of care for patients with breast cancer.

Also presented at ASCO 2008 was a retrospective study that evaluated bevacizumab's tolerance in the elderly patient population.[29] The study examined the medical record of patients above the age of 60 with MBC who were treated with bevacizumab combination therapy. The study suggested that these older patients had more grade 3/4 thrombosis, bleeding, perforation, fatigue and febrile neutropenia than patients in historical randomized phase III trials of bevacizumab plus chemotherapy. It was concluded that the risks and benefits of bevacizumab therapy must be weighed prior to its use in the elderly population.

## Hormonal-resistance reversing agents

The majority of breast cancers in post menopausal women express estrogen and/or progesterone receptors. This subgroup of breast cancers carries a better prognosis than estrogen/progesterone receptor negative patients, and they can often be treated with hormonal therapy alone. However hormone resistance ultimately becomes a major treatment barrier and, cytotoxic chemotherapy becomes a necessity. There is evidence that estrogen receptor positive breast cancers become resistant to hormonal therapy by up-regulating other signaling pathways involved in tumor proliferation such as EGFR, HER2, MAPK and PI3K/Akt. [30-32] Novel strategies have now been employed to overcome this resistance by the addition of new signal transduction inhibiting agents to standard hormonal agents.

### Gefitinib

Gefitinib is an orally-active small molecule selective EGFR tyrosine kinase inhibitor. Its use as monotherapy in advanced and refractory MBC has proven to be disappointing as demonstrated by two early phase II clinical trials for which it was found to have little anti-tumor activity in breast cancer.[33,34] However, its activity in combination therapy has shown more potential. It has been evaluated in combination therapy in two phase II studies for which it was combined with docetaxel in the first-line setting. In the first study, 41 patients received oral gefitinib 250 mg per day along with docetaxel at 75 mg/m2 and 100 mg/m2.[35] There was no difference in activity or tolerability between the two docetaxel doses. The ORR was 54% with a complete response (CR) and partial response (PR) in 22 out of 41 patients. Toxicities included neutropenia, diarrhea, rash and anemia.

The second study was a phase II multi-institutional trial to determine the efficacy and tolerability of gefitinib 250 mg daily and docetaxel 75 mg/m2 as first-line treatment in patients with MBC presented at ASCO 2007.[36] 33 patients with MBC received the combination of gefitinib and docetaxel, and the results demonstrated a CBR of 51% and an ORR of 39.4%. Most toxicities were attributed to docetaxel rather than gefitinib. It was concluded that the combination of docetaxel and gefitinib was an active regimen in MBC, and the toxicities and efficacy were similar to those of docetaxel alone. Unfortunately, gefitinib's activity in MBC could not be elucidated in these two phase II studies as the trials did not include a docetaxel alone group for comparison.

At ASCO 2008, gefitinib was evaluated in combination with anastrozole in a phase II multicenter, double blind, randomized trial to investigate its efficacy on reversing resistance to hormone therapy.[37] In this trial, 94 women with newly diagnosed hormone receptor positive MBC were randomized to receive anastrozole 1 mg daily in combination with either gefitinib 250 mg daily or placebo with the primary end point of the study being PFS. The results of the study showed a superior PFS in the gefitinib group when compared with placebo (14.5 months vs 8.2 months). The CBR also favored the gefitinib group when compared with placebo (49% vs 34%). The treatment-related adverse events were generally mild and well tolerated but were seen twice as often in the gefitinib arm when compared with the placebo arm. It was concluded that the combination of anastrazole plus gefitinib is well tolerated and shows increased anti-tumor activity when compared to anastrazole alone in this group of MBC patients. These results are promising and demands further study of this combination in MBC patients.

### RAD001

RAD001 is a highly specific inhibitor of the mammalian target of rapamycin (mTOR), a large polypeptide kinase which forms part of the PI3K/Akt pathway. This pathway is a central regulator of intracellular signaling pathways involved in tumor cell growth, proliferation and angiogenesis.[38,39] Its use is currently gaining attention in the treatment of several genitourinary malignancies, but its use in breast cancer has also been investigated. A phase I study investigated the safety and pharmacokinetics of combined treatment with letrozole 2.5 mg per day and RAD001 at at 5 mg or 10 mg per day in patients with MBC stable or progressing after > or = 4 months on letrozole alone.[40] Seven patients received the combination therapy for > 6 months. One patient had a complete response, and one had a 28% reduction in liver metastases, both in the high dose RAD001 group. The most common adverse events were stomatitis, fatigue, anorexia and/or decreased appetite, diarrhea, headache and rash. There was no clinically relevant pharmacokinetic interaction detected between the two agents. It was concluded that therapy with RAD001 plus letrozole is promising with the overall safety profile of the combination consistent with that expected for RAD001 monotherapy.

This study led the way to a study presented at ASCO 2008 for which RAD001 was investigated in combination with letrozole in a randomized phase II trial in ER positive breast cancer patients in the neoadjuvant setting.[41] 270 post-menopausal women with ER positive tumors were randomized to letrozole 2.5 mg daily plus RAD001 10 mg daily or letrozole plus placebo. The clinical response rate favored the combination of letrozole plus RAD001 arm over the letrozole plus placebo arm (68% vs 59%). However, the rate of high grade toxicity was more frequent in the combination group when compared with placebo (22.6% vs 3.8%). The most common adverse events were hyperglycemia, stomatitis, interstitial lung disease and infections. It was concluded that RAD001 significantly increases the efficacy of letrozole in newly diagnosed ER positive breast cancer.

At 2008 ASCO, RAD001 was also evaluated in a phase I study where it was combined with weekly paclitaxel 80 mg/m2 and trastuzumab 2 mg/kg weekly in patients with HER2 positive MBC with prior resistance to trastuzumab.[42] At the time of the presentation, 13 heavily pretreated patients were enrolled and treated with paclitaxel and trastuzumab with the addition of RAD001 given in a daily or weekly basis. All 3 patients in the daily arm achieved a PR. Of the 4 patients in the weekly arm, 2 patients achieved a PR, 1 patient achieved a minor regression, and 1 patient achieved stable disease. The most common adverse events were neutropenia and stomatitis. The combination was well tolerated and showed probable activity in this group of heavily pretreated patients.

In a second phase I study presented at ASCO 2008, RAD001 was combined with vinorelbine and trastuzumab in a similar group of patients.[43] In this study, vinorelbine 25 mg/m2 and trastuzumab 2 mg/kg weekly was combined with RAD001 given in a weekly or daily fashion and administered to 19 heavily pretreated HER2 positive patients who had progressed on trastuzumab therapy. Of the 8 evaluable patients in the daily arm, 2 patients achieved a PR, and 4 patients achieved SD. Toxicities included stomatitis and neutropenia. Of the 9 evaluable patients in the weekly arm, 1 patient achieved a PR and 7 patients achieved stable disease. Neutropenia was the most common adverse event reported in this group of patients. It was concluded that RAD001 is well tolerated in combination with vinorelbine and trastuzumab and shows potential anti-tumor activity in heavily pretreated HER2 positive MBC patients. The trial continues to accrue at this time.

## Other agents

### Tyrosine kinase inhibitors

#### Pazopanib

Pazopanib is an oral, multi-targeted inhibitor targeting all isoforms of VEGFR, platelet-derived growth factor receptor (PDGFR) and c-kit. It has already shown to be efficacious in renal cell cancer (RCC) in a phase II randomized discontinuation trial in 225 patients with metastatic RCC.[44] At ASCO 2008, a randomized study compared pazopanib 400 mg daily plus lapatinib 1000 mg daily to lapatinib 1500 mg daily alone in patients with untreated HER2 positive advanced or MBC in the first-line setting.[45] The progressive disease rate was higher in the lapatinib alone group when compared to the combination group (27% vs 19%). The ORR also favored the combination group (44%) when compared to the lapatinib monotherapy group (30%). The most common adverse effects in the combination group were diarrhea, rash and nausea. Liver function abnormalities were also more common in the combination group. This was the first phase II trial to evaluate the combination of two oral targeted agents in first-line HER2 positive MBC patients.

#### Sunitinib

Sunitinib is an orally-active small molecule tyrosine kinase inhibitor that acts on multiple targets including VEGFR, PDGFR, c-kit and Flt-3.[46] In a phase II, open-label, multicenter study, sunitinib was evaluated as monotherapy in patients with MBC. 64 patients previously treated with an anthracycline and a taxane received sunitinib 50 mg daily in six week cycles, and results showed that 7 patients achieved a partial response with a median duration of 19 weeks giving an ORR of 11%. 3 patients achieved stable disease for greater than 6 months. The median TTP was 10 weeks, and the OS was 38 weeks. The most common adverse events were fatigue, nausea, diarrhea, mucosal inflammation and anorexia, but most were mild to moderate and effectively managed.

These promising results have led to many ongoing clinical trials using sunitinib in the metastatic setting. One trial will evaluate the combination of sunitinib with docetaxel in patients with MBC).[47] A second trial will evaluate sunitinib in combination with docetaxel and traztuzumab in advanced HER2 positive MBC patients).[47] Lastly, There is a neoadjuvant study using sunitinib, approved by NCI and will be open for accrual shortly through SWOG. This trial is designed as a phase II study using weekly nanoparticle albumin bound paclitaxel (nab-paclitaxel), with or without sunitinib followed or preceded by weekly doxorubicin and daily cyclophosphamide as neoadjuvant therapy for inflammatory and locally advanced Her-2/Neu negative breast cancer. These upcoming trials may lead to the incorporation of sunitinib in future regimens in not only the metastatic setting but also the neoadjuvant setting as well.

#### Axitinib

Axitinib (AG013736) is an orally active multi-kinase inhibitor that inhibits the receptor tyrosine kinases VEGFR 1 and 2, PDGFR and c-KIT. Its activity in breast cancer was demonstrated at ASCO 2007 in a randomized double blind phase II study of axitinib 5 mg twice daily in combination with docetaxel 80 mg/m2 or placebo.[48] 168 previously untreated patients with MBC were randomized in this study, and the results showed a significant ORR in favor of the axitinib group when compared to placebo (40% vs 23%). In addition, TTP favored the axitinib group when compared to placebo (9 months vs 6.3 months). The most common adverse events in the combination arm were diarrhea, nausea, alopecia, fatigue and stomatitis. It was concluded that the combination of axitinib and docetaxel is tolerable and has potential anti-tumor activity for MBC in the first-line setting. Further studies using axitinib in breast cancer are ongoing.

### Topoisomerase II inhibitors

#### C1311

C1311 is an inhibitor of topoisomerase II whose design was based upon mitoxantrone but with less cardiotoxicity. At 2008 ASCO, a phase II trial including 53 MBC patients resistant to taxanes, anthracyclines, multiple hormonal therapies and other cytotoxic agents were treated with C1311 480 mg/m2 weekly.[49] The ORR was found to be 40% with 36% of patients achieving stable disease. The main toxicity was neutropenia, and cardiac toxicity was minimal. It was concluded that C1311 shows activity in pretreated MBC with some disease control and a manageable safety profile. Further studies including C1311 are ongoing.

### Bisphosphonates

#### Zoledronic Acid

Bisphosphonate use in breast cancer was also examined in several studies at ASCO 2008. Zoledronic acid has demonstrated anti-tumor and anti-metastatic activity in preclinical models, and it was investigated in an ABCSG-12 study evaluating adjuvant ovarian suppression combined with tamoxifen or anastrozole alone or in combination with zoledronic acid in premenopausal women with endocrine-responsive stage I and II breast cancer.[50] 1,801 premenopausal women with endocrine-responsive breast cancer were administered either goserelin 3.6 mg q 28 days and tamoxifen 20 mg daily with or without zoledronic acid 4 mg iv q 6 months or goserelin and anastrazole 1 mg daily with or without zoledronic acid for a period of three years. There was no significant difference in results for patients who received tamoxifen or anastrazole alone, but anti-hormonal therapy with zoledronic acid increased disease free survival by 36% and relapse-free survival by 35% compared to anti-hormonal therapy alone. The difference in OS was not significant but trended in favor of the zoledronic acid arms. It was concluded that adjuvant zoledronic acid improves outcomes in endocrine responsive patients further than anti-hormonal therapy alone.

A second study evaluated the effects of zoledronic acid on bone mineral density comparing upfront versus delayed treatment in an open-label trial in postmenopausal women with primary breast cancer starting letrozole after tamoxifen.[51] 558 women without osteoporosis were treated with zoledronic acid 4 mg q 6 months either in an upfront or a delayed fashion, and the results demonstrated that patients in the upfront arm averaged a 3.7% increase in lumbar spine bone mineral density while the delayed arm averaged a 1.7% decrease in bone mineral density. The rates of clinically meaningful decline in lumbar spine bone mineral density also favored the upfront arm when compared to the delayed arm (3% vs 20.7%). It was concluded that upfront use of zoledronic acid significantly prevented bone loss when compared to delayed therapy in postmenopausal patients without osteoporosis starting letrozole following tamoxifen for primary breast cancer.

#### Ibandronate

Ibandronate is another bisphosphonate that was evaluated at ASCO 2008. In a double blind, randomized, placebo controlled trial labeled the ARIBON trial, the effect of ibandronate on bone mineral density was assessed.[52] 131 post menopausal women were categorized into three groups: women with normal bone density, women with osteopenia, and women with osteoporosis. All patients were treated with anastrozole 1 mg daily, calcium and vitamin D. The patients in the osteopenic group were randomized in a 1:1 fashion to either ibandronate or placebo. The patients in the osteoporosis group all received ibandronate 150 mg monthly. The patients in the osteopenia group who received ibandronate achieved a mean difference in percentage bone mineral density changes of 6.2% at the lumbar spine and 4.5% at the hip after 2 years of therapy when compared to placebo. Improvement in bone mineral density was also observed in the group of patients with osteoporosis when treated with ibandronate. It was concluded that oral ibandronate prevents anastrozole-induced bone loss and results in significant increases in bone mineral densities at the lumbar spine and the hip in this group of osteopenic and osteoporotic patients.

These studies not only reinforce the necessity of bisphosphonate therapy in patients receiving endocrine therapy for the prevention of bone loss, but they also illustrate the powerful anti-tumor activity of these agents as well.

### Novel chemotherapies

#### Pemetrexed

Pemetrexed is a multi-targeted anti-folate that inhibits several enzymes in the de novo synthesis of purines and pyrimidines. Several studies have examined its efficacy in MBC beginning with a phase II study in patients with locally recurrent or MBC. In this study, 38 MBC patients were treated with pemetrexed 600 mg/m2, and the ORR was 28% with one patient achieving a CR and 9 patients achieving a PR. Median duration of response was 9 months and median OS was 13 months. Toxicities included neutropenia and thrombocytopenia.[53] This study led to another phase II study involving pemetrexed in the treatment of MBC patients pretreated with anthracyclines.[54] In this study, 77 patients were treated with pemetrexed 600 mg/m2, and the ORR was found to be 21%. Median duration of response was 5.5 months, and median OS was 10.7 months. Again, high grade toxicities included neutropenia and thrombocytopenia. Another phase II study evaluated pemetrexed in heavily pretreated MBC.[55] The patients involved in the study were previously treated with an anthracycline, a taxane and capecitabine for MBC. 80 patients were treated with pemetrexed 600 mg/m2, and the ORR was found to be 8% with stable disease achieved in 36% of patients with a median OS of 8.2 months.

Another study explored the efficacy and toxicity of pemetrexed treatment at two different doses.[56] The study was a phase II randomized, double-blind study using a 600 mg/m2 and 900 mg/m2 doses of pemetrexed to treat locally recurrent or MBC in the first-line setting. The ORR was similar in the two groups with the low dose group achieving an ORR of 17% and the high dose group achieving an ORR of 15.6%. The PFS was also similar in both groups (4.2 months vs 4.1 months). Both arms exhibited minimal toxicity. The study confirmed that both doses of pemetrexed yielded similar response rates and toxicity profiles.

Pemetrexed has also been evaluated in combination therapy. In a phase II open label, multi-center study of pemetrexed and carboplatin, 50 patients with locally advanced or MBC were treated in the first-line setting with the combination of pemetrexed 600 mg/m2 and carboplatin AUC 5.[57] The ORR was found to be 54% with a median response duration of 11.1 months and a median time to disease progression of 10.3 months. Toxicities were predominantly bone marrow suppression related. It was concluded that the combination of pemetrexed plus carboplatin was feasible and had promising activity in the first-line setting for the treatment of MBC.

At ASCO 2008, pemetrexed was evaluated as first-line therapy for advanced or MBC patients.[58] 37 patients with advanced or MBC were treated with pemetrexed 600 mg/m2, and the results revealed an ORR of 26.5% with 47% of patients achieving stable disease. The median duration of response was 6.5 months, and the median PFS was 4.1 months. Median OS was 18.9 months. Again, toxicities were related to myelosuppression and anemia. The study showed that pemetrexed achieved moderate activity in the first-line setting with a tolerable toxicity profile without alopecia.

#### Larotaxel

Larotaxel (XRP9881) is a novel taxoid with preclinical activity against taxane-resistant breast cancer. It first gained attention in a phase II study in patients with MBC who previously received taxane-based therapy.[59] In this study139 patients were stratified by response to prior taxane therapy (resistant or non-resistant) and received larotaxel 90 mg/m2 monotherapy. In the non-resistant group, the ORR was 42%, the median duration of response was 5.3 months, the median TTP was 5.4 months and the median survival time was 22.6 months. In the resistant group the ORR was 19%, the median duration of response was 5 months, the median TTP was 1.6 months, and the median survival time was 9.8 months. The most common adverse events were neutropenia and fatigue. It was concluded that larotaxel has activity in patients refractory to taxane treatment with a tolerable safety profile.

At ASCO 2008, larotaxel was evaluated in combination with trastuzumab in patients with HER2 positive MBC.[60] The patients received larotaxel 90 mg/m2 and trastuzumab 6 mg/kg every three weeks. The presentation was an interim analysis of a phase II open label study in HER2 positive MBC patients that included patients with asymptomatic brain metastases. Out of 26 evaluable patients, 11 patients (42.3%) achieved a PR, and the most common adverse events were neutropenia, diarrhea, and asthenia. This study suggests that the combination of trastuzumab and larotaxel is feasible with good activity in pretreated patients with high tumor burden including brain metastases.

#### Ortataxel

Ortataxel is another novel new-generation taxane which has shown activity in preclinical models in tumors resistant to taxane therapy. At 2008 ASCO, a single-arm phase II study was presented treating taxane-resistant MBC with ortataxel 75 mg/m2.[61] Of the 76 evaluable patients, 7% of patients achieved a PR while 38% achieved stable disease. The most common adverse events were neutropenia, peripheral neuropathy, fatigue and malaise. It was concluded that ortataxel continues to have activity in taxane-resistant MBC patients and has a manageable toxicity profile.

## Future therapies

Several other agents continue to be investigated for the treatment of breast cancer patients that appear to be promising. One compound that was evaluated at ASCO 2008 was paclitaxel polyglumex which is a macromolecular conjugate of paclitaxel bound to poly-L-glutamic acid which appears more attractive than traditional paclitaxel in that it causes less alopecia, has a shorter infusion time, requires no premedication, and has an enhanced tumor permeability.[62] The agent was combined with capecitabine and used to treat MBC patients in a single stage phase II study and was found to be tolerable and have activity in MBC patients.

IMP321 is a novel immunomodulator derived from the natural human protein LAG-3, a ligand for MHC class II molecules which acts indirectly on T cell responses by MHC class II APC activation.[63] It was evaluated in combination with weekly paclitaxel in a phase I study in MBC at ASCO 2008 and was found to be well tolerated when given subcutaneously over 6 months.

BZL101 is an aqueous extract of the sutellaria barbata herb which demonstrated in vitro growth inhibiting properties in breast cancer cell lines without affecting normal breast cancer cells thus having a favorable toxicity profile. It was evaluated at 2008 ASCO in a phase I open-label study of heavily pretreated MBC patients receiving BZL101 in a dose escalating fashion.[64] Of 5 patients evaluable for response, one patient achieved stable disease for 8 months with radiographic evidence of tumor shrinkage. The most common adverse events included diarrhea, nausea, headache and ALT increase. It was concluded that BZL101 has a favorable toxicity profile with encouraging activity in heavily pretreated MBC patients.

Eribulin mesylate is a non-taxane microtubule dynamics inhibitor which was evaluated at ASCO 2008 in a phase II study in MBC patients previously treated with anthracycline, taxane, and capecitabine therapy.[65] The single arm, open label phase II study enrolled 291 patients, and the results showed an ORR of 9.3% and a CBR of 17.1% in this group of heavily pretreated patients.

Enzastaurin is a potent serine-threonine kinase inhibitor which selectively targets PKCβ and PI3K/AKT signaling pathways and was evaluated at ASCO 2008 in a phase II study in MBC patients previously treated with an anthracycline and a taxane containing regimen.[66] The monotherapy was well tolerated but it was found to have no activity in this pretreated population of MBC patients.

PF-00299804 is an orally active pan-HER tyrosine kinase inhibitor which specifically and irreversibly binds to and inhibits HER1, HER2 and HER4 which was evaluated in non-small cell lung cancer patients at ASCO 2008 but also exhibits potential activity in HER2 positive breast cancer patients.[67]

Motesanib is a small molecule tyrosine kinase inhibitor of VEGF that targets VEGFR 1, 2, 3 and PDGF that has been evaluated in preclinical models in combination with either tamoxifen or docetaxel and was shown to have potential use in breast cancer.[68] A large phase II clinical trial is ongoing evaluating motesanib and paclitaxel in the treatment of breast cancer).[47]

Vorinostat is a histone deacetylase inhibitor that is currently approved for treatment of cutaneous T-cell lymphoma but has also shown activity in other malignancies.[69] It is currently being evaluated in the treatment of MBC in combination therapy with multiple agents including tamoxifen and capecitabine.[69]

GDC-0449 is a novel small molecule antagonist of the hedgehog signaling pathway which is an important pathway for tumor survival and growth implicated in multiple solid organ tumors.[70] It was evaluated at ASCO 2008 in a first in human, first in class phase I study in patients with advanced solid tumors and hopes to be a promising agent in the treatment of breast cancer.[71]

## Conclusion

In recent years there have been significant advances in the treatment of all types of cancer, but in particular breast cancer. These accomplishments have been led by the arrival of targeted therapies including hormonal inhibitors, HER2 inhibitors and now inhibitors of several additional targets. Many of these agents are now incorporated in the first-line treatment of several groups of MBC patients and continue to be the foundation of successful treatments. In addition, the manipulation of conventional chemotherapeutic agents for a more pronounced effect in MBC has been an exciting area in the treatment of breast cancer.

With increasing tumor resistance to chemotherapy in MBC, it is of vital importance to continue to apply these novel therapies to current treatment standards and to continue to search for newer agents that are under investigation for use in breast cancer. The role of most of these biologic and targeted agents is thus far undefined. The relatively safe toxicity profile and convenience of these agents often times makes them a more attractive option when compared to conventional cytotoxic chemotherapy. It is now imperative for researchers and clinicians to establish the efficacy and role of these novel agents in combination or as monotherapy in order to develop new strategies in the treatment of breast cancer. By continuing to evaluate these agents in clinical trials, investigators hope to not only define their role in cancer therapeutics but also stimulate a further influx of targeted therapies in the future.

## Competing interests

The authors declare that they have no competing interests.

## References

[B1] National Cancer Institute (2008). Surveillance Epidemiology and End Results. http://www.seer.cancer.gov.

[B2] Blackwell K, Kaplan H, Franco S, Marcom P (2004). A phase II, open-label, multicenter study of GW572016 in patients with trastuzumab-refractory metastatic breast cancer. Journal of Clinical Oncology Annual ASCO meeting.

[B3] Gomez H, Chavez M, Doval D (2005). Biomarker results from a phase II randomized study of lapatinib (GW572016) as first-line treatment for patients with ErbB2 FISH-amplified advanced or metastatic breast cancer. Breast Cancer Research and Treatment.

[B4] Kaufman B, Trudeau M, Johnston S, Awada A Clinical activity of lapatinib monotherapy in patients with HER2+ relapsed/refractory inflammatory breast cancer (IBC): Final results of the expanded HER2+ cohort in EGF103009. journal of Clinical Oncology annual ASCO meeting 2008.

[B5] Geyer C, Forster J, Lindquist D, Chan S (2006). Lapatinib plus capecitabine for HER2-positive advanced breast cancer. New England Journal of Medicine.

[B6] Di Leo A, Gomez H, Aziz Z, Zvirbule Z Lapatinib with paclitaxel compared to paclitaxel as first-line treatment for patients with metastatic breast cancer: A phase III randomized, double-blind study of 580 patients. Journal of Clinical Oncology, 2007 ASCO Annual Meeting, Abstract #1011(No 18S).

[B7] O'Shaughnessy J, Blackwell K, Burstein H, Storniolo A A randomized study of lapatinib alone or in combination with trastuzumab in heavily pretreated HER2 positive metastatic breast cancer progressing on trastuzumab therapy. Journal of Clinical Oncology Annual ASCO meeting 2008, abstract #1015(44s).

[B8] Rugo H, Franco S, Munster P, Stopeck A A phase II evaluation of lapatinib and bevacizumab in HER2 positive metastatic breast cancer. Journal of Clinical Oncology Annual ASCO meeting 2008, abstract #1042(51s).

[B9] Rabindran S, Discafani C, Rosfjord E (2004). Antitumor activity of HKI-272, an orally active, irreversible inhibitor of the HER-2 tyrosine kinase. Cancer Res.

[B10] Burstein H, Awada A, Badwe R (2007). HKI-272, an irreversible pan erbB receptor tyrosine kinase inhibitor: preliminary phase 2 results in patients with advanced breast cancer. Breast Cancer Research and Treatment.

[B11] National Cancer Institute (2008). http://www.cancer.gov.

[B12] Osipo C, Patel P, Hao L (2007). ErbB-2 inhibition activates notch-1 and sensitizes breast cancer cells to a gamma-secretase inhibitor: opportunity for a novel therapeutic combination. Breast Cancer Res Treat.

[B13] Beeram M, Burris H, Modi S, Birkner M A phase I study of trastuzumab-DM-1, a first in class HER2 antibody-drug conjugate, in patients with advanced HER2+ breast cancer. Journal of Clinical Oncology Annual ASCO meeting 2008, abstract #1028(48s).

[B14] Holden S, Beeram M, Krop I, Burris H A phase I study of weekly dosing of trastuzumab-DM1 in patients with advanced HER2+ breast cancer. Journal of Clinical Oncology 2008 Annual ASCO meeting, abstract #1029(48s).

[B15] Cortes L, Mayer E, Marcom P (2005). Open label, randomized, phase II study of pertuzumab in patients with metastatic breast cancer with low expression of HER2. Journal of Clinical Oncology.

[B16] Gelmon K, Furnoleau P, Verma S, Wardley A Results of a phase II trial of trastuzumab and pertuzumab in patients with HER2 positive metastatic breast cancer who had progressed during trastuzumab therapy. Journal of Clinical Oncology annual ASCO meeting 2008, abstract #1026(47s).

[B17] Zsebik B, Citri A, Isola J (2006). Hsp90 inhibitor 17-AAG reduces ErbB2 levels and inhibits proliferation of the trastuzumab resistant breast tumor cell line JIMT-1. Immunol Lett.

[B18] Modi S, Sugarman S, Stopeck A, Linden H Phase II trial of the Hsp90 inhibitor tanespimycin plus trastuzumab in patients with HER2 positive metastatic breast cancer. journal of Clinical Oncology annual ASCO meeting 2008, abstract #1027(47s).

[B19] O'Shaughnessy J, Weckstein D, Vukelja S (2007). Preliminary results of a randomized phase II study of weekly irinotecan/carbopatin with or without cetuximab in patients with metastatic breast cancer. Breast Cancer Research and Treatment.

[B20] Carey l, Rugo H, Marcom P, Irvin W TBCRC 001: EGFR inhibition with cetuximab added to carboplatin in metastatic triple negative (basal-like) breast cancer. journal of Clinical Oncology annual ASCO meeting 2008, abstract #1009(43s).

[B21] Hobday T, Stella P, Fitch T, Jaslowski A N0436: A phase II trial of irinotecan plus cetuximab in patients with metastatic breast cancer and prior anthracycline and/or taxane-containing therapy. Journal of Clinical Oncology annual ASCO meeting 2008, abstract #1081(61s).

[B22] Cobleigh M, Langmuir V, Sledge G (2003). A phase I/II dose-escalation trial of bevacizumab in previously treated metastatic breast cancer. Semin Oncolgoy.

[B23] Miller K, Wang M, Gralow J (2005). A randomized phase III trial of paclitaxel versus paclitaxel plus bevacizumab as first-line therapy for locally recurrent or metastatic breast cancer. A trial coordinated by the Eastern Cooperative Oncology Group (E2100). 28th Annual San Antonio Breast Cancer Symposium.

[B24] Miller K, Chap L, Holmes F (2005). Randomized phase III trial of capecitabine compared with bevacizumab plus capecitabine in patients with previously treated metastatic breast cancer. Journal of Clinical Oncology.

[B25] Miles D, Chan A, Romieu G, Dirix L Randomized, double-blind, placebo-controlled, phase III study of bevacizumab with docetaxel or docetaxel with placebo as first-line therapy for patients with locally recurrent or metastatic breast cancer (mBC): AVADO. Journal of Clinical Oncology annual ASCO meeting 2008, abstract #LBA1011(43s).

[B26] Danso M, Blum J, Robert N, Krekow L Phase II trial of weekly nab-paclitaxel in combination with bevacizumab as first-line treatment in metastatic breast cancer. journal of Clinical Oncology annual ASCO meeting 2008, abstract #1075(59s).

[B27] Hurvitz A, Bosserman L, Leland-Jones B, Thirwell M A multicenter, double-blind randomized phase II trial of neoadjuvant treatment with single-agent bevacizumab or placebo, followed by docetaxel, doxorubicin, and cyclophosphamide, with or without bevacizumab, in patients with stage II or stage III breast cancer. Journal of Clinical Oncology Annual ASCO meeting, abstract # 562(21s).

[B28] Pierga J, Pritchard K, Cortes-Funes H, Biganzoli L M019391: An open-label safety study of bevacizumab plus taxane-based therapy as first-line treatment of patients with locally recurrent or metastatic breast cancer. Journal of Clinical Oncology Annual ASCO meeting, abstract #1140(76s).

[B29] Richardson S, Dickler M, Dang C, Hudis C Tolerance of bevacizumab in an older patient population: THe Memorial Sloan-Kettering Cancer Center experience. journal of Clinical Oncology annual ASCO meeting 2008, abstract #9569(519s).

[B30] Tokunaga E, Kimura Y, Masino K (2006). Activation of PI3K/Akt signalling and hormone resistance in breast cancer. Breast Cancer.

[B31] Kurokawa H, Lenferink A, Simpson J (2000). Inhibition of HER2/neu and mitogen-ativated protein kinases enhances tamoxifen action against HER2-overexpressing, tamoxifen-resistant breast cancer cells. Cancer Res.

[B32] Jeng M, Yue W, Eischeid A (2000). Role MAP kinase in the enhanced cell proliferation of long term estrogen deprived human breast cancer cells. Breast Cancer Research and Treatment.

[B33] Baselga J, Albanell J, Ruiz A (2005). Phase II and tumor pharmacodynamic study of gefitinib in patiens with advanced breast cancer. Journal of Clinical Oncology.

[B34] Von Minckwitz G, Jonat W, Fasching P (2005). A multicenter phase II study on gefitinib in taxane- and anthracycline-pretreated metastatic breast cancer. Breast Cancer Research and Treatment.

[B35] Ciardiello F, Troiani T, Caputo F, De Laurentiis M (2006). Phase II study of gefitinib in combination with docetaxel as first-line therapy in metastatic breast cancer. British Journal of Cancer.

[B36] Dennison S, Jacobs S, Wilson J, Seeger J (2007). A phase II clinical trial of ZD1839 (Iressa) in combination with docetaxel as first-line treatment in patients with advanced breast cancer. Invest New Drugs.

[B37] Cristofanilli M, Valero V, Mangalik A, Rabinowitz I A phase II multicenter, double blind, randomized trial to compare anastrozole plus gefitinib with anastrazole plus placebo in postmenopausal women with hormone receptor positive metastatic breast cancer. Journal of Clinical Oncology annual ASCO meeting 2008, abstract #1012(44s).

[B38] Schmelzle T, Hall M (2000). TOR, a central controller of cell growth. Cell.

[B39] Yu Y, Sato J (1999). MAP kinases, phosphatidylinositol 3-kinase, and p70 S6 kinase mediate the mitogenic response of human endothelial cells to vascular endothelial growth factor. J Cell Physiology.

[B40] Awada A, Cardoso F, Fontaine C, Dirix L (2008). The oral mTOR inhibitor RAD001 (everolimus) in combination with letrozole in patients with advanced breast cancer: results of a phase I study with pharmacokinetics. Eur J Cancer.

[B41] Baselga J, van Dam P, Greil R, Gardner H Improved clinical and cell cycle response with an mTOR inhibitor, daily oral RAD001 (everolimus) plus letrozole versus placebo plus letrozole in a phase II neoadjuvant trial in ER+ breast cancer. Journal of Clinical Oncology annual ASCO meeting 2008,abstract #530(13s).

[B42] Andre F, Campone M, Hurvitz S, Vittori L Multicenter phase I clinical trial of daily and weekly RAD001 in combination with weekly paclitaxel and trastuzumab in patients with HER2-overexpressing metastatic breast cancer with prior resistance to trastuzumab. Journal of Clinical Oncology annual ASCO meeting 2008, abstract #1003(41s).

[B43] Jerusalem G, Dieras V, Cardoso F, Bergh J Multicenter phase I clinical trial of daily and weekly RAD001 in combination with vinorelbine and trastuzumab in patients with HER2-overexpressing metastatic breat cancer with prior resistance to trastuzumab. Journal of Clinical Oncology annual ASCO meeting 2008, abstract #1057(55s).

[B44] Hutson T, Davis I, Machiels J, de Souza P Pazopanib (GW786034) is active in metastatic renal cell carcinoma: interim results of a phase II randomized discontinuation trial. Journal of Clinical Oncology ASCO meeting 2007, abstract #5031.

[B45] Slamon D, Gomez H, Kabbinavar F, Amit O Randomized study of pazopanib + lapatinib vs lapatinib alone in patients with HER2 positive advanced or metastatic breast cancer. Journal of Clinical Oncology annual ASCO meeting 2008, abstract #1016(45s).

[B46] Burstein H, Elias A, Rugo H (2008). Phase II study of sunitinib malate, an oral multitargeted tyrosine kinase inhibitor, in patients with metastatic breast cancer previously treated with an anthracycline and a taxane. Journal of Clinical Oncology.

[B47] National Institute of health (2008). http://www.clinicaltrials.gov/ct/show/.

[B48] Rugo H, Stopeck A, Joy A, Chan S A randomized, double-blind phase II study of the oral tyrosine kinase inhibitor axitinib (AG013736) in combination with docetaxel compared to docetaxel plus placebo in metastatic breast cancer. Journal of Clinical Oncology annual ASCO meeting 2007, abstract #1003(18s).

[B49] Capizzi R, Roman L, Tjulandin S, Smirnova I Phase II trial of C1311, a novel inhibitor of topoisomerase II in advanced breast cancer. Journal of Clinical Oncology annual ASCO meeting, 2008, abstract #1055(54s).

[B50] Gnant M, Mlineritsch B, Schippinger W, Luschin-Ebengreuth G Adjuvant ovarian suppression combined with tamoxifen or anastrozole, alone or in combination with zoledronic acid, in premenopausal women with hormone-responsive, stage I and II breast cancer: First efficacy results from ABCSG-12. Journal of Clinical Oncology ASCO meeting 2008, Abstract #LBA4(1006s).

[B51] Mincey B, Dentchev T, Sloan J, Hines S N03CC–a randomized, controlled, open-label trial of upfront vs. delayed zoledronic acid for prevention of bone loss in postmenopausal (PM) women with primary breast cancer (PBC) starting letrozole after tamoxifen. journal of Clinical Oncology annual ASCO meeting 2008, Abstract #LBA4(1006s).

[B52] Lester J, Dodwell D, Purohit O, Gutcher S Use of monthly oral ibandronate to prevent anastrozole-induced bone loss during adjuvant treatment for breast cancer: Two-year results from the ARIBON study. Journal of Clinical Oncology annual ASCO meeting 2008, abstract #554(19s).

[B53] Miles D, Smith I, Coleman R, Calvert A (2001). A phase II study of pemetrexed disodium (LY2311514) in patients with locally recurrent or metastatic breast cancer. Eur J Cancer.

[B54] Martin M, Spielmann M, Namer M, duBois A (2003). Phase II study of pemetrexed in breast cancer patients pretreated with anthracyclines. Annals of Oncology.

[B55] O'shaughnessy J, Clark R, Blum J, Mennel R (2005). Phase II study of pemetrexed in patients pretreated with an anthracycline, a taxane, and capecitabine for advanced breast cancer. Clinical Breast Cancer.

[B56] Llombart-Cussac A, Martin M, Harbeck N, Anghel R (2007). A randomized, double blind, phase II study of two doses of pemetrexed as first-line chemothearpy for advanced breast cancer. Clinical Cancer Research.

[B57] Garin A, Manikhas A, Biakhov M, Chezhin M (2008). A phase II study of pemetrexed and carboplatin in patients with locally advanced or metastatic breast cancer. Breast Cancer Research and Treatment.

[B58] Robert N, Conkling P, Kuefler P, McIntyre K Results of a phase II study of pemetrexed as first-line chemotherapy for advanced or metastatic breast cancer. Journal of Clinical Oncology annual ASCO meeting 2008, abstract#1073(59s).

[B59] Dieras V, Limentani S, Romieu G, Tubiana-Hulin M (2008). Phase II multicenter study of larotaxel (XRP9881), a novel taxoid, in patients with metastatic breast cancer who previously received taxane-based therapy. Annals of Oncology.

[B60] Dieras V, Viens P, Veyret C, Romieu G Larotaxel in combination with trastuzumab in patients with HER2 positive metastatic breast cancer: Interim analysis of an open label phase II study. Journal of Clinical Oncology annual ASCO meeting 2008, abstract #1070(58s).

[B61] Beer M, Lenaz L, Amadori D Phase II study of ortataxel in taxane-resistant breast cancer. Journal of Clinical Oncology annual ASCO meeting 2008, abstract #1066(57s).

[B62] Northfelt D, Allred J, Liu H, Hobday T Phase II trial of paclitaxel polyglumex (PPX) with capecitabine (C) for metastatic breast cancer (MBC). Journal of Clinical Oncology annual ASCO meeting 2008, abstract #1063(56s).

[B63] Triebel F, Brignone C, Grygar, Marcu M IMP321 and weekly paclitaxel as first-line chemoimmunotherapy for metastatic breast cancer (MBC). Journal of Clinical Oncology annual ASCO meeting 2008, abstract#1045(52s).

[B64] Perez A, Shaw H, Fleming G, Hershman D A phase I trial of scutellaria barbata (BZL101) for metastatic breast cancer. journal of Clinical Oncology annual ASCO meeting 2008, abstract #1099(65s).

[B65] Vahdat L, Twelves C, Allison M, Cortes J Phase II study of eribulin mesylate (E7389) in patients (pts) with locally advanced or metastatic breast cancer (MBC) previously treated with anthracycline, taxane, and capecitabine therapy. Journal of Clinical Oncology annual ASCO meeting 2008, abstract#1084(62s).

[B66] Krop I, Miller K, Zon R, Isakoff S A phase II study of oral enzastaurin in patients with metastatic breast cancer previously treated with an anthracycline and a taxane-containing regimen: HOG BRE05-97. Journal of Clinical Oncology annual ASCO meeting 2008, abstract #1004(42s).

[B67] Janne P, Schellens J, Engelman J, Eckhardt Preliminary activity and safety results from a phase I clinical trial of PF-00299804, an irreversible pan-HER inhibitor, in patients (pts) with NSCLC. Journal of Clinical Oncology annual ASCO meeting, 2008, abstract#8027(430s).

[B68] Coxon A, Bush T, Belmontes B Anti-tumor activity of motesanib diphosphaate alone and in combination with docetaxel or tamoxifen in xenogrft models of human breast carcinoma. Breast Cancer Research and Treatment, 106(abstract #1102): s75.

[B69] Glaser, Keith (2007). HDAC inhibitors: Clinical update and mechanism-based potential. Biochemical Pharmacology.

[B70] Rubin L, Sauvage F (2006). Targeting the hedghog pathway in cancer. Nat Rev Drug Disc.

[B71] LoRusso P, Rudin C, Boarad M, Vernillet L A first-in-human, first-in-class, phase (ph) I study of systemic Hedgehog (Hh) pathway antagonist, GDC-0449, in patients (pts) with advanced solid tumors. Journal of Clinical Oncology annual ASCO meeting, 2008, abstract #3516(157s).

